# Fístulas da Artéria Coronária

**DOI:** 10.36660/abc.20210501

**Published:** 2021-07-15

**Authors:** Dario Buccheri

**Affiliations:** 1 Unidade de Cardiologia IntervencionistaS. Antonio Abate HospitalTrapaniItáliaUnidade de Cardiologia Intervencionista, S. Antonio Abate Hospital,Trapani - Itália

**Keywords:** Isquemia Miocárdica, Fístula Arterio-Arterial/diagnóstico por imagem, Fístula Arterio-Arterial/cirurgia, Angiografia Coronária/métodos, Técnicas de Fechamento/tendências, Antibacterianos/uso terapêutico

## Abstract

A fístula da artéria coronária é uma anormalidade anatômica rara das artérias coronárias que afeta 0,002% da população geral e representa 14% de todas as anomalias das artérias coronárias. A sua relevância clínica concentra-se principalmente no mecanismo do fenômeno do roubo coronário, que causa isquemia funcional do miocárdio, mesmo na ausência de estenose; portanto, angina e dispneia aos esforços são sintomas comuns. A abordagem diagnóstica sugerida é orientada pelos sintomas dos pacientes e consiste em uma série de exames instrumentais, como ECG, teste de esteira, ecocardiografia, tomografia computadorizada, ressonância magnética cardíaca e angiografia coronária. Nos casos onde não é um achado acidental, a angiografia coronária é necessária para o planejamento terapêutico otimizado. As pequenas fístulas geralmente são assintomáticas e o prognóstico é excelente se forem tratadas medicamente com acompanhamento clínico e ecocardiografia no período de 2 a 5 anos. As fístulas grandes/gigantes e sintomáticas, ao contrário, devem ser submetidas a fechamento invasivo, por via transcateter ou ligadura cirúrgica, cujos resultados são equivalentes no acompanhamento de longo prazo. A profilaxia antibiótica para a prevenção da endocardite bacteriana é recomendada para todos os pacientes com fístulas da artéria coronária submetidos a procedimentos dentários, gastrointestinais ou urológicos. O acompanhamento ao longo da vida é sempre essencial para garantir que o paciente não sofra progressão da doença ou outras complicações cardíacas.

A fístula da artéria coronária (FAC) é uma conexão entre uma ou mais artérias coronárias e uma câmara cardíaca (fístula coronária-cameral) ou um vaso sanguíneo maior (fístula arteriovenosa) quando o leito capilar do miocárdio é desviado. Embora sejam geralmente isolados (80%), também podem estar associados a outras malformações cardíacas congênitas (20%), incluindo tetralogia de Fallot, persistência do canal arterial, e defeitos do septo atrial e do septo ventricular.^1,2^

A incidência exata de FAC ainda é desconhecida, pois a taxa de casos não diagnosticados permanece alta, mas estima-se que, enquanto a incidência de anomalias coronárias é de 0,2% a 1,2% na população geral, a FAC está presente em 0,002%.^3,4^ A FAC representa cerca de 0,2% a 0,4% de todas as malformações cardíacas^5^ e 14% de todas as anomalias coronárias.^6^ Diversos estudos indicam FAC em 0,3% dos pacientes que apresentavam cardiopatia congênita, em 0,06% das crianças submetidas à ecocardiografia e em 0,13% a 0,22% dos adultos submetidos à angiografia coronária.^7^

Cerca de 75% de todos os casos de FAC descobertos acidentalmente são pequenos e clinicamente silenciosos.^8^

Embora, no passado, a etiologia da FAC fosse predominantemente das formas congênitas, ao longo dos anos, o desenvolvimento e a disseminação de técnicas intervencionistas e cirúrgicas têm resultado em mudanças na sua etiologia, com maior prevalência das formas adquiridas,^9^ que podem incluir formas secundárias a endocardite infecciosa, dissecção aórtica, cirurgia anterior, biópsia endomiocárdica, angioplastia coronária, cirurgia de revascularização, substituição valvar, transplante cardíaco, trauma, colocação de marca-passo permanente, ablação de vias acessórias com tórax fechado, neoplasias e manejo iatrogênico de doença de Kawasaki.^10^

A artéria que alimenta a fístula pode drenar de uma artéria coronária ou de um de seus ramos e, geralmente após um curso dilatado e tortuoso, termina em uma das câmaras cardíacas ou em um vaso. A FAC com origem proximal é frequentemente grande; pelo contrário, se a sua origem é distal, geralmente é menor e mais tortuosa.^11^

Pode haver múltiplas artérias alimentando um único ponto de drenagem da FAC ou podem existir múltiplos locais de drenagem. Também foram relatadas múltiplas fístulas entre as 3 principais artérias coronárias e o ventrículo esquerdo. Em alguns casos, especialmente em adultos, as fístulas podem originar-se de ambas as artérias coronárias, que drenam para o tronco pulmonar. Essas fístulas podem, frequentemente, causar angina e exigir o fechamento.^12^ A FAC surge com mais frequência da artéria coronária direita (aproximadamente 50% a 60%) e drena com mais frequência no coração direito (aproximadamente 80%).

As fístulas de grande calibre podem permitir o conhecido fenômeno do roubo coronário, no qual o sangue com escoamento diastólico é direcionado para longe da circulação coronária normal e da microcirculação miocárdica. Quando o local de drenagem está localizado no átrio esquerdo ou na veia pulmonar, há efetivamente um shunt esquerdo-esquerdo que determina uma sobrecarga de volume apenas para o coração esquerdo.

Ocasionalmente, pode ser realizado um diagnóstico presuntivo ao ouvir um sopro atípico sistólico, diastólico ou contínuo, embora os sinais possam ser encontrados no ECG, na radiografia de tórax ou na ecocardiografia.

Os exames esclarecedores são a tomografia computadorizada com múltiplos detectores ou a ressonância magnética. A tomografia computadorizada é superior à ecocardiografia em pacientes com sobrepeso e permite um excelente delineamento anatômico, ao contrário da ecocardiografia. A angiografia coronária continua sendo a melhor técnica diagnóstica para a detecção de FAC com envolvimento estrutural cardíaco e para avaliação hemodinâmica; além disso, possibilita programar o fechamento intervencionista com dispositivos dedicados.^13^

As FAC de tamanho pequeno ou moderado devem ser fechadas apenas no caso em que os pacientes são sintomáticos para isquemia miocárdica, arritmias, dilatação ventricular ou disfunção de origem incerta, ou se houver complicações por endocardite. Caso contrário, os pacientes com fístulas pequenas e assintomáticas não precisam ser submetidos a fechamento, apenas a acompanhamento clínico com ecocardiografia no período de 2 a 5 anos.

Pacientes com FAC submetidos a fechamento percutâneo ou cirúrgico têm bom prognóstico, podendo variar de acordo com as possíveis complicações relacionadas às técnicas, a gravidade do shunt e a morfologia da fístula. A expectativa de vida, no entanto, é normal, com taxas de recorrência variando de 9% a 19% para o fechamento transcateter e 25% para a ligadura cirúrgica.^2,6,14^

Mais detalhes estão agora disponíveis graças a recentes estudos interessantes.^15^
