# Supplementary material for: Fístulas da Artéria Coronária
Source: Arq Bras Cardiol. 2021 Jul 15;117(1):89–90. [Article in Portuguese] doi: 10.36660/abc.20210501 (PMC8294720; doi:10.36660/abc.20210501)
Supplement: Translation

# Translation: Coronary Artery Fistulae

**Authors:** Dario Buccheri

**Affiliations:** 1 ItalyUnidade de Cardiologia Intervencionista, S. Antonio Abate Hospital,Trapani - Italy

**Keywords:** Myocardial Ischemia, Arterio-arterial fistula/diagnostic imaging, Arterio-arterial fistula/surgery, Coronary Angiography/methods, Closure Techniques/trends, Anti-Bacterial Agents/therapeutic use

## Abstract

Coronary artery fistula is a rare anatomic abnormality of the coronary arteries that affects 0.002% of the general population and represents 14% of all anomalies of coronary arteries. Its clinical relevance focuses mainly on the mechanism of the coronary steal phenomenon, causing myocardial functional ischemia, even in the absence of stenosis; therefore, angina and effort dyspnea are common symptoms. The suggested diagnostic approach is driven by patients’ symptoms, and it consists of a number of instrumental examinations like ECG, treadmill test, echocardiography, computed tomography scan, cardiac magnetic resonance, and coronary angiography. If it is not an incidental finding, coronary angiography is required in view of optimal therapeutic planning. Small fistulae are usually asymptomatic, and prognosis is excellent if they are managed medically with clinical follow-up and echocardiography every 2 to 5 years. Large/giant, symptomatic fistulae, on the contrary, should undergo invasive closure, via either transcatheter approach or surgical ligation, whose results are equivalent at long-term follow-up. Antibiotic prophylaxis for prevention of bacterial endocarditis is recommended in all patients with coronary artery fistulae who undergo dental, gastrointestinal, or urological procedures. Life-long follow-up is always essential to ensure that the patient does not undergo progression of the disease or further cardiac complications.

Coronary artery fistula (CAF) is a connection between one or more coronary arteries and a cardiac chamber (coronary-cameral fistulae) or a major blood vessel (arteriovenous fistulae) when the myocardial capillary bed is bypassed. While they are generally isolated (80%), they may also be associated with other congenital cardiac malformations (20%) including tetralogy of Fallot, patent ductus arteriosus, atrial septal defects, and ventricular septal defects.^1,2^

The exact incidence of CAF is still unknown, because the rate of undiagnosed cases remains high, but it is estimated that, whereas the incidence of coronary anomalies is 0.2% to 1.2% in the general population, CAF is present in 0.002%.^3,4^ CAF represents about 0.2% to 0.4% of all cardiac malformations^5^ and 14% of all coronary anomalies.^6^ Several other studies indicate CAF in 0.3% of patients who presented with congenital heart disease, in 0.06% of children undergoing echocardiography, and in 0.13% to 0.22% of adults undergoing coronary angiography.^7^

About 75% of all CAF that are discovered incidentally are small and clinically silent.^8^

Although, in the past, the etiology of CAF was prominently of the congenital forms, over the years, the development and dissemination of interventional and surgical techniques have resulted in changes to its etiology, with a higher prevalence of the acquired forms,^9^ which may include those secondary to infective endocarditis, aortic dissection, previous surgery, endomyocardial biopsy, coronary angioplasty, bypass surgery, valve replacement, cardiac transplant, trauma, permanent pacemaker placement, closed-chest ablation of accessory pathways, neoplasms, and iatrogenic management of Kawasaki disease.^10^

The feeding artery of the fistula may drain from a coronary artery or one of its branches, and, usually after a dilated and tortuous course, it ends in one of the cardiac chambers or a vessel. CAF with a proximal origin is frequently large; on the contrary, if its origin is distal, it is usually tinier and more tortuous.^11^

There may be multiple feeding arteries to a single CAF drainage point, or multiple drainage sites may exist. Multiple fistulae between the three major coronary arteries and the left ventricle have also been reported. In some cases, especially in adults, fistulae may originate from both coronary arteries, which drain into the pulmonary trunk. These fistulae can frequently cause angina and require closure.^12^ CAFs arise more frequently from the right coronary artery (approximately 50% to 60%) and drain most often in the right heart (approximately 80%).

Fistulae with a large caliber could allow the well-known phenomenon of coronary steal in which the blood with a diastolic runoff is directed away from the normal coronary circulation and myocardial microcirculation. When the drainage site is located in the left atrium or pulmonary vein, there is an effective left-to-left shunt that determines a volume overload to the left heart only.

A presumptive diagnosis can occasionally be made upon hearing an atypical systolic, diastolic, or continuous murmur, although signs could be found on ECG, chest X-ray, or echocardiography.

Clarifying exams are multidetector computed tomography or magnetic resonance imaging . Computed tomography is superior to echocardiography in patients who are overweight, and they allow excellent anatomical delineation, in contrast to echocardiography. Coronary angiography remains the best diagnostic technique for CAF detection with cardiac structural involvement and for hemodynamic evaluation; furthermore, it makes it possible to program interventional closure with dedicated devices.^13^

CAF of small or moderate size should be closed only in the event that patients are symptomatic for myocardial ischemia, arrhythmias, ventricular dilation, or dysfunction of uncertain origin, or if there are complications due to endocarditis. Otherwise, patients with small, asymptomatic fistulae should not be subjected to closure, but they should undergo clinical follow-up with echocardiography every 2 to 5 years.

Patients with CAF who undergo percutaneous or surgical closure have good prognosis, which depends on the possible complications related to the techniques, the severity of the shunt, and the morphology of the fistula. Life expectancy is, however, normal, with recurrence rates ranging from 9% to 19% for transcatheter closure and 25% in surgical ligation.^2,6,14^

Further details have just been made available thanks to recent interesting studies.^15^
